# Detection of Dynamic Spatiotemporal Response to Periodic Chemical Stimulation in a *Xenopus* Embryonic Tissue

**DOI:** 10.1371/journal.pone.0014624

**Published:** 2011-01-31

**Authors:** YongTae Kim, Sagar D. Joshi, William C. Messner, Philip R. LeDuc, Lance A. Davidson

**Affiliations:** 1 Departments of Mechanical Engineering, Biomedical Engineering, and Biological Sciences, Carnegie Mellon University, Pittsburgh, Pennsylvania, United States of America; 2 Departments of Bioengineering and Developmental Biology, University of Pittsburgh, Pittsburgh, Pennsylvania, United States of America; University of California Merced, United States of America

## Abstract

Embryonic development is guided by a complex and integrated set of stimuli that results in collective system-wide organization that is both time and space regulated. These regulatory interactions result in the emergence of highly functional units, which are correlated to frequency-modulated stimulation profiles. We have determined the dynamic response of vertebrate embryonic tissues to highly controlled, time-varying localized chemical stimulation using a microfluidic system with feedback control. Our approach has enabled localized spatiotemporal manipulation of the steroid hormone dexamethasone (DEX) in Animal Cap (AC) tissues isolated from gastrulating *Xenopus* embryos. Using this approach we investigated cell-scale responses to precisely controlled stimulation by tracking the redistribution of a GFP-tagged DEX-reporter constructed from the human glucocorticoid receptor (GR). We exposed defined regions of a single AC explant to different stimulation conditions—continuous stimulation, periodic stimulation, and no stimulation. We observed collective behavior of the GR transport into the nucleus was first-order. Furthermore, the dynamic response was well-modeled by a first-order differential equation with a single time derivative. The model predicted that responses to periodic stimulations closely matched the results of the frequency-based experiments. We find that stimulation with localized bursts versus continuous stimulation can result in highly distinct responses. This finding is critical as controlled space and time exposure to growth factors is a hallmark of complex processes in embryonic development. These complex responses to cellular signaling and transport machinery were similar to emergent behaviors in other complex systems, suggesting that even within a complex embryonic tissue, the overall system can converge toward a predictive first-order response.

## Introduction

Embryonic development is a complex, dynamic and highly regulated feedback process where cells actively respond to and exert control over their environment to form intact tissues, which results in functioning organ systems [1], [2]. These regulatory interactions lead to the emergence of highly functional units, which are correlated to frequency controlled stimulation profiles [3]. Tissues develop and mature by integrating signaling information provided by several internal and external cues such as genes, mechanical and architectural cues, and growth factors in the form of gradients [4]-[7]. During development, gradients of diffusible chemical growth factors and morphogens play a fundamental role in the feedback control processes that shapes animal form [8]–[10]. Since these chemical gradients direct cell differentiation into specific tissue types and guide cell migration to specific locations [11], [12], controlling these gradients will be a key requirement when engineering complex tissues for organ regeneration.

Embryonic tissues from developing embryos such as the fruit fly (*Drosophila melanogaster*), the nematode worm (*Caenorhabditis elegans*), and vertebrates such as the house mouse (*Mus musculus*), the zebrafish (*Danio rerio*), and the clawed frog (*Xenopus laevis*) have been used extensively to identify factors and the molecular pathways that transduce chemical stimuli into cellular responses [13]. One classical approach adopted by experimental embryologists is to use excised tissue fragments, or explants, microsurgically removed from *Xenopus* embryos to study localized developmental processes [14]. These classical approaches have been complemented by more modern tools to visualize cells and analyze gene and protein expression [15]–[17].

Control of the stimulation profile within microsurgically-isolated tissues serves to reduce the potential complexity of chemical stimuli operating within developing multicellular embryos. For example, chemical gradients can be controlled by manipulating the micro-environment [18], [19], delivering growth factors or modulating their activity, such as overexpressing growth factors to level the gradient or saturate receptors or genes encoding inhibitory factors or dominant negative receptors [20]. These approaches have been key tools in identifying factors that induce differentiation of a range of tissues and testing their physiological function within live embryos [21]. However, it is hard to regulate the chemical activity of factors delivered by beads or overexpression. Therefore, the ability to deliver regulators of embryonic development with long-term spatiotemporal control will provide the more sophisticated regulation needed to engineer organs and tissues *ex vivo*.

Our microfluidic implementation with feedback regulation overcomes many limitations of manual approaches, allowing investigation of both rapid biological responses such as those seen during calcium signaling [22], and long term responses needed during organ formation [23]. Many conventional microfluidic approaches rely on commercial syringe pumps. Combined with automated feedback control, these tools can be used to probe short-term events such as occurring during calcium signaling dynamics [24], [25]. However, the drawback of these approaches is that they are not well-suited for long-term dynamic manipulation of microfluidic laminar flow in time and space [26], [27]. Here, we utilize a novel pressure modulation mechanism with feedback control [28] to examine developmental signaling processes where long-term kinetics of time- and space-varying responses in multicellular tissues can be captured.

Here we examine the response of *Xenopus laevis* AC explants isolated from gastrulating embryos to a chemical environment precisely controlled by microfluidics. Using spatial and temporal microfluidic control we engineer three distinct microenvironments in a single AC explant where we can compare patterns of a hormone biosensor translocation into the nucleus in response to continuous and periodic hormone stimulation. We find we can model this translocation with a first-order transport equation and analyze the responses to temporally regulated complex stimuli in a systematic manner. The results indicate that close examination of the system-based response to frequency-based stimulation highlights a process that contributes to directing embryonic tissue responses to their intricate chemical microenvironment.

## Results

### Biosensor Construction

**Figure 1:**
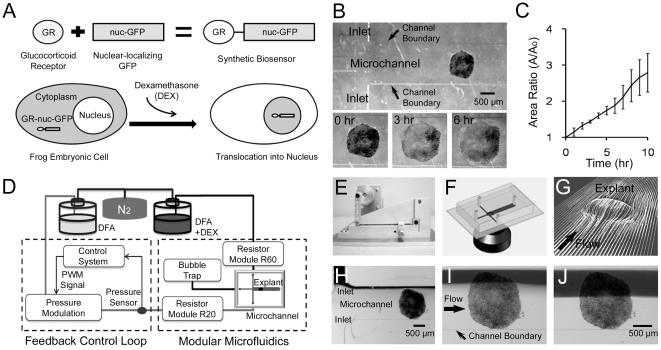
Spatiotemporal control of dexamethasone over *Xenopus* Animal Cap explants with the biosensor GR-nuc-GFP using a pressure feedback microfluidic approach. (A) Construction of DEX biosensor GR-nuc-GFP. GR-nuc-GFP resides in the cytoplasm, but moves into the nucleus after DEX is added. Dark areas in the cells indicate accumulation of GR-nuc-GFP. (B) Tissue explants from different frogs attached to the substrate in the microfluidic channels. Tissue explants spreading at the begining (left panel), 3 hours (middle panel), and 6 hours (right panel) after attachment in the microfluidic channel. (C) Ratio of the area of the tissue explants normalized by the initial area versus time (n = 3). Error bars represent standard deviations. (D) Microfluidic interface control system consisting of feedback control loop and modular microfluidics (see Materials and Methods). (E) Pressure modulation mechanism that allows long-term and high-speed control of the flow rate in a microfluidic channel [28]. (F) Schematic showing the confocal microscopic imaging of the cross-typed microfluidic channel. (G) Simulations showing flow pathlines over and around a single explant through a Computational Fluid Dynamics (CFD) simulation indicating no flow disruption around the explant. (H) Laminar flow interface between the stream of DEX (upper inlet; black) and the stream DFA (lower inlet) before the experiment. (I) Regulated laminar flow interface covers quarter of the AC explant. (J) Laminar flow interface moves to the center of the channel, exposing DEX to the half of the AC explant.

To probe the kinetics of cellular responses within a multicellular embryonic tissue to chemical stimulation, we first created a synthetic stimulation-response network using the human glucocorticoid response system expressed within embryonic AC explants (Figure 1A). To detect activation by the glucocorticoid hormone dexamethasone (DEX) we constructed a GFP-based biosensor that reports the level of hormone stimulation in *Xenopus* cells by fusing the hormone binding domain from the human glucocorticoid receptor (GR) [29] with a nuclear-localizing green fluorescent protein containing a nuclear import sequence (nuc-GFP) [30], [31]. AC explants expressing the biosensor (GR-nuc-GFP) show that GFP fluorescence initially accumulates in cytoplasm in the absence of DEX and translocates into the nucleus after the addition of DEX to the system. We tested the effectiveness of this reporter by collecting confocal stacks of the AC explant cultured in conventional chambers at 0, 60, and 120 minutes after addition of DEX. GR-nuc-GFP moved into the nucleus less than 60 minutes after addition of DEX (Figure S1). We confirmed that GR-nuc-GFP moved into the nucleus by fixing AC explants and co-staining their nuclei with propidium iodide (Figure S2). We also demonstrated our ability to track this biosensor by monitoring the dynamics of individual cells in the AC explant expressing GR-nuc-GFP and calculating the ratio of GFP intensity within the nucleus and the cytoplasm in tracked cells at 30 minute intervals (Figure S3).

### Embryonic Cell Spreading in a Microchannel

To test the health of AC explants in the microfluidic channel we followed their development with low magnification time-lapse microscopy. Explants were first attached to the glass coverslip that were the bottom surface of the microfluidic channel (Figure 1B). After the attachment, the explants spread for more than 6 hours (Figure 1B: Video S1). At times typically more than 6 hours, the edges of the explants approached the walls of the 1.5 mm wide channel. The area covered by explants can increase three-fold over 10 hours (Figure 1C). Explants spreading beyond the edge of the channel can perturb fluid flow in unpredicatable ways. For example, our CFD simulations predicted unexpectedly nonlinear flow streamlines as once the explant spans the channel. Therefore, we typically began experiments three hours after AC explants were loaded within the microfluidic channel and completed experiments before explants spanned the channel.

### Feedback microfluidic control with the biosensor enabled

With GR-nuc-GFP as a DEX biosensor we followed internal responses of cells within the embryonic tissue (i.e. a live cell “output” of the internal functional state of the system) to a precise spatiotemporal pattern of DEX stimulation (i.e. controlled the “input” to the system). We used our microfluidic approach to control stimulation over the large area spanned by a typical AC explant and provide time-varying stimulation over longer durations. Thus we had control over localized stimulation and the ability to monitor spatiotemporal responses over the entire explant. Our microfluidic control system (Figure 1D), using a custom designed pressure regulation mechanism (Figure 1E; [28]) delivered precise doses of DEX to tightly defined regions; stacks of optical sections were taken using time lapse confocal microscopy (Figure 1F). Integrating the biosensor with microfluidics and confocal imaging enabled both long-term and high-speed manipulation of DEX environments and allowed the readout of the cell-by-cell response within embryonic *Xenopus laevis* AC explants.

Although flow is strictly laminar at low Reynolds number, it is important to consider the effect of the three-dimensional (3D) shape of the tissue on flow patterns and the diffusion of chemical factors across the laminar flow interface. The Reynolds number is a dimensionless number indicating the ratio of inertial to viscous forces in fluid mechanics [32]; a Reynolds number less than 1 implies a viscous flow field such as those produced within microfluidic channels while a large Reynolds number indicates inertial forces can dominate the flow field and lead to turbulence. We theoretically examined the contribution of these factors to create a functioning system through modeling the fluid interactions with the geometry of a 3D tissue using computational fluid dynamics (CFD) simulations (Figure S4), revealing that no flow disruption develops around the explant in the experiment (Figure 1G). CFD simulations can provide limits on the range of exploitable flow rates critical to precise stimulation [33], [34]. We then experimentally determined the highest flow rate of approximately 50 µl/min in the condition of our microfluidic channel that would not shear the AC explant attached to the substrate. In order to maintain laminar flow with minimal diffusion we determined the lowest flow rate of approximately10 µl/min. With these ranges of the flow rate, the calculated Reynolds number remained less than 1 (Figure S4C, D). These experimental and simulation studies dictated a flow rate of 30 µl/min for all subsequent experiments. To achieve this flow rate required an inlet pressure of 2 kPa for the resistance of the microfluidic channel (Figure S4D). This flow rate corresponded to a fluid velocity around the explant of less than approximately 2 mm/s and a shear rate of less than 30 s^−1^ (Figure S4E); this fluid velocity and shear rate are very small when compared to the rates used with either whole embryos or dissociated cells [34], [35].

We also considered the possible diffusion of chemical factors across the laminar flow interface. A flow rate of 30 µl/min limits diffusive dispersion across the laminar interface to approximately 15 µm based on CFD simulations where the determination of diffusion was based on a 10% threshold of mass fraction (Figure S4C). This flow regime is characterized by a Peclet number above 3000 [32]. The Peclet number is a dimensionless number indicating the ratio of the rate of advection to diffusion; a small Peclet number indicates excessive diffusive mixing while a large Peclet number indicates a sharp interface between the streams. To verify laminar flow under these conditions, we recorded time-lapse sequences of fluid flow around the AC explant. Flow around the AC explant was strictly laminar with a linear or planar interface between the streams (Figure 1H). Thus at these flow rates sharp laminar flow interfaces between DEX (black stream) and DFA could be positioned at desired locations within the microchannel without disruption due to the 3D shape of the tissue (Figure 1I, J).

### Response to spatially patterned stimulation

**Figure 2:**
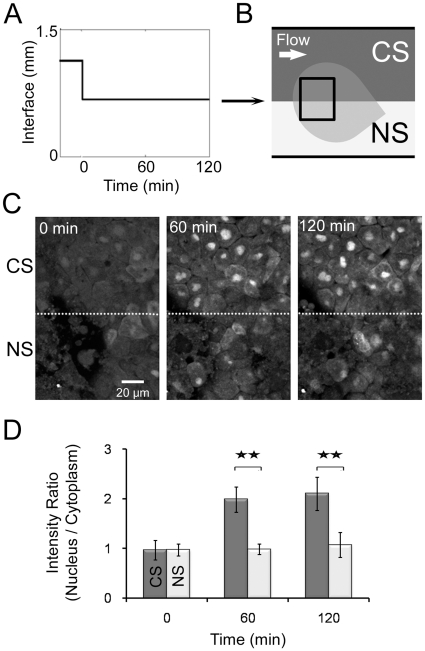
Localized response to spatially defined continuous stimulation. (A) Laminar flow interface profile over time. (B) Schematic depicting two regions within a single AC explant subject to different stimulation conditions: constant stimulation (CS; upper region; DEX & DFA) and no stimulation (NS; lower region; DFA). (C) Images of the explant subjected to CS and NS depicted in (B) at the beginning (left), 60 minutes (middle), and 120 minutes (right). The dotted line marks the interface, which correlates to the line between CS and NS regions in (B). (D) Intensity ratios of GFP levels in nucleus relative to cytoplasm. Error bars represent standard deviations for 20 cells (** indicates p<0.01). Variable expression of GR-nuc-GFP biosensor across the animal cap is due to the uneven inheritance of mRNA encoding GR-nuc-GFP into 1 or 2-cell stage embryo.

To investigate responses to a sharp gradient we positioned the interface between the streams of DFA and DEX over the center of an explant for 120 minutes (Figure 2A) and collected image stacks at 60 minute intervals using confocal microscopy. One region of the explants was exposed to DEX continuously (continuous stimulation or CS) while the other was only exposed to flow with DFA (no stimulation or NS; Figure 2B). NS and CS regions showed significantly different degrees of translocation of GR-nuc-GFP (Figure 2C). We found no apparent translocation of GR-nuc-GFP in the explant at the beginning of the experiment (left panel, Figure 2C), but after 60 minutes, the constantly stimulated (CS) regions exhibited a stronger nuclear localization of GR-nuc-GFP than the non-stimulated (NS) regions (middle panel, Figure 2C); the trend continued over 120 minutes (right panel, Figure 2C). We analyzed the intracellular responses by calculating the ratio of the GFP intensity in the subcellular nuclear region divided by the intensity in the subcellular cytoplasmic region within the same cell (Figure 2D). Quantitatively, CS regions of the explant showed a significantly high intensity ratio at 60 minutes (91% greater, Figure 2D) than the NS regions and reached to a steady state level by 120 minutes.

### Response to spatiotemporally patterned stimulation

**Figure 3:**
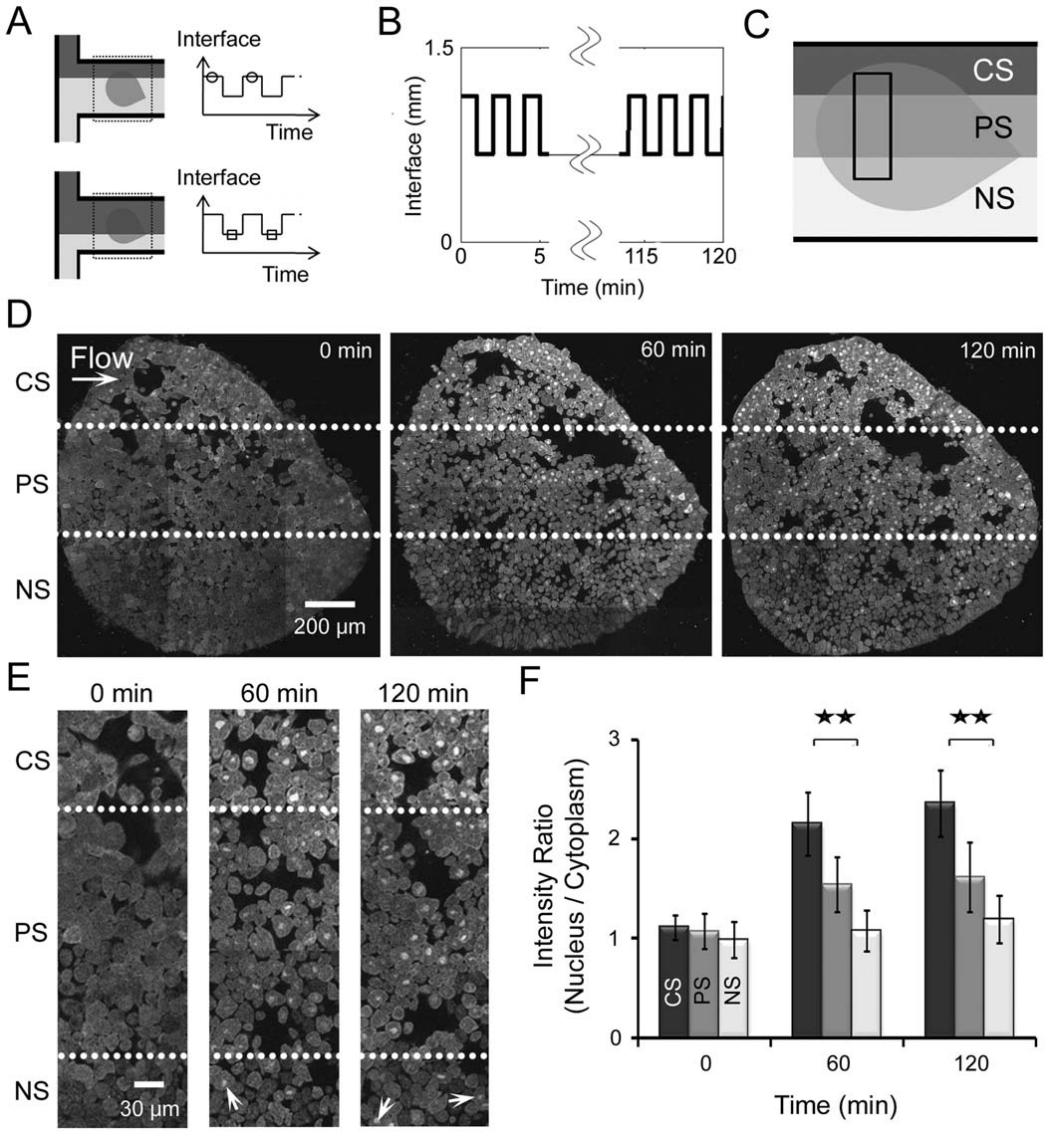
Localized responses to spatiotemporal periodic stimulations with 50% duty cycles. (A) The stream of DEX is controlled by directing a laminar flow interface over the explant allowing periodic stimulation profiles to be applied. Circles on the top of the periodic pattern of the interface represent initial interface positions while squares on the bottom of the pattern indicate repositioned interfaces. (B) Laminar flow interface profile over time. (C) Schematic depicting three regions of a single AC explant exposed to different stimulation conditions: CS (upper region), 2-minute 50% duty cycle PS (middle), and NS (lower). (D) AC explants exposed to CS, PS, and NS regions of (C) at 0 minutes (left), 60 minutes (middle), and 120 minutes (right). The dotted lines mark the interfaces, which correlate to the lines between CS, PS, and NS regions in (C). (E) High resolution views of explants shown in (D) in the area indicated by the rectangular shape in (C) at the beginning (left), 60 minutes (middle), and 120 minutes (right). The dotted lines represent the interfaces, which correlate to the lines between CS, PS, and NS regions in (C) and (D). (F) Intensity ratios of GFP in the nucleus versus cytoplasmic intensities. Error bars represent standard deviations for 20 cells (** indicates p<0.01). Variable expression of GR-nuc-GFP biosensor across the animal cap is due to the uneven inheritance of mRNA encoding GR-nuc-GFP into 1 or 2-cell stage embryo.

Simple forms of frequency stimulation such as “pulse-chase” experiments have been used to explore the role of long range factors in developing embryos [11]. To investigate the developmental response of an integrated embryonic tissue to complex signals we applied frequency controlled stimulation to a single AC explant (Figure 3A) and collected image stacks at 60 minute intervals using confocal microscopy. We began by testing tissue responses to a 2-minute periodic flow profile with a 50% duty cycle (we define the duty cycle as the fraction of the period where the localized region of the explant is exposed to DEX) for 120 minutes over the center region (periodic stimulation or PS) while maintaining CS and NS over other regions (Figure 3B, C). Responses to continuous stimulation (CS) and no stimulation (NS) were qualitatively similar to earlier experiments (e.g. Figure 2C, D) at the beginning, 60 minutes, and 120 minutes after stimulation (Figure 3D). For instance, cells in the CS region exhibited significant increases in the number of GFP-labeled nuclei at both 60 and 120 minutes (upper sector, Figure 3D). In contrast to CS regions, some cells in PS regions showed apparent translocation while others exhibited little response (middle sector, Figure 3D). Cells in NS regions had very few changes in intensities (lower sector, Figure 3D). A higher magnification image provided more spatial details on events at the critical interfacial region (Figure 3E, the rectangular region of Figure 3C). Almost all of the cells in CS regions exhibit high intensity of the GR-nuc-GFP in nucleus over time while the PS cells either show less intensity or no response (Figure 3E). Cell responses within regions exposed to DEX were not all binary (i.e. “on” nor “off”) but rather they had higher or lower nuclear intensities compared to the cytoplasm, indicating cells had variable responses in these regions. We note occasional cells in the NS regions had GR-nuc-GFP in the nucleus (see arrows in NS regions at 60 minutes (middle) and 120 minutes (right) in Figure 3E). These cells do not necessarily indicate leaking DEX since we have observed occasional cells with spontaneously localized GFP in the nucleus even in explants that have never been exposed to DEX (see arrowheads in the AC explant cultured in conventional chambers without DEX as a control experiment in Figure S1D). We suspect two possible explanations for the translocation of GR-nuc-GFP into the nucleus in the absence of DEX. One possibility is that a small number of cells might inherit a high concentration of mRNA from the earlier injection. Through trial-and-error we have injected 0.2 ng of mRNA encoding GR-nuc-GFP for each frog embryo. There is always some variability in the expression level viewed with a confocal microscope. At very high levels of expression, typically 5-fold more than we inject, we observe spontaneously localized GFP. We speculate that exogenous GR may bind all available heat shock proteins (HSPs) and some GR-domains are "free" of HSP allowing their translocation into the nucleus. Another possibility is that some cells have increased use of HSP by other cellular processes, driving HSP from GR-binding sites in the cells that are not exposed to DEX. However, the number of cells with GFP spontaneously translocating to the nucleus is small and their presence does not alter our overall kinematic analysis. These spontaneously translocated reporters have been observed in previous studies with cultured cells [36]. We quantified cell responses by normalizing the nuclear-to-cytoplasm GFP intensity ratios to the initial pre-DEX ratios (Figure 3F). As exhibited in the images (Figure 3D, E), cells in CS and NS regions showed qualitatively similar responses to earlier experiments (Figure 2D) at 0, 60, and 120 minutes after stimulation while the PS cells exhibited approximately half of the intensity ratio responses when compared to the CS cells. Through this approach, we were able to apply continuous and periodic stimulation with our microfluidic control system to elicit spatially distinct responses.

### Response to temporally patterned stimulation

**Figure 4:**
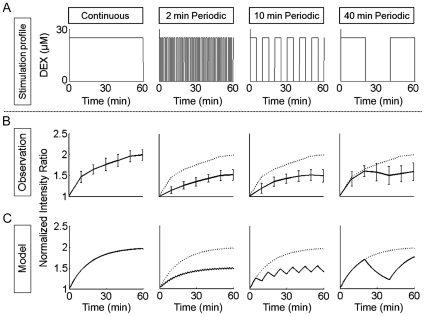
Responses of the tissue explant to four different stimulation profiles: continuous stimulation (CS), and 50% duty cycle periodic stimulation (PS); 2 min-, 10 min-, and 40 min-period. (A) Input stimulation profiles. (B) Responses of 30 individual tracked cells from 3 different tissue explants to four different stimulation cases with different duty cycles: CS, 2-minute 50% duty cycle PS, 10-minute 50% duty cycle PS, and 40-minute 50% duty cycle PS. Error bars indicate standard deviations. Dotted lines represent the response to CS (left panel). (C) Mathematical model recapitulated GR-nuc-GFP movements after various periodic stimulation profiles. This model was constructed using a first-order differential equation (see Materials and Methods). The parameters reproducing the response to CS were applied to the other PS cases to predict their response without any additional parameters (modeled CS; modeled 2-minute PS; modeled 10-minute PS; and modeled 40-minute PS). Dotted lines represent the modeled response to CS (left panel). The modeled results well approximate experimental results in (B).

To investigate the dynamics of the nuc-GR signaling and nuclear import system we applied a more complex program of stimulation. We wondered whether an explant responds the same to 50% stimulation as it would to alternating between zero and 100% for equal amounts of time; or whether an explant exposed to 100% stimulation followed by an “off” cycle simply resumes its response at the same level when the 100% stimulation is reapplied. We created four different DEX stimulation profiles: continuous stimulation, and 2 min-, 10 min-, and 40 min-periodic stimulations with 50% duty cyles (Figure 4A). We tracked the responses of a larger population of 30 individual cells from 3 explants (10 cells in each explant; Figure 4B) exposed to these four different stimulation profiles every 10 minutes over 60 minutes. The response to the 2-minute and 10-minute periodic stimulations with 50% duty cycles was about half of the response of the continuous stimulation. In contrast, the response to the 40-minute periodic stimulation with 50% duty cycle was approximately the same as the response of the continuous stimulation for the first 20 minutes (the “on” part of the period, Figure 4A, B) and then decreased between 20 minutes to 40 minutes (the “off” part of the period, Figure 4A, B). The response slightly increased again between 40 minutes and 60 minutes (the “on” part of the second period, Figure 4A, B); however, these increases were not statistically significant. In general, the long-term responses to stimulation profiles approach constant levels (Figure 4B; see also Figure S6). In addition to transport into the nucleus, GR-nuc-GFP can move out of the nucleus over a longer time period (a half-time of ∼4 hrs, [36]) after the DEX is washed out (Figure S7); however, export is considerably slower than import [36]. Thus, we conclude that responses to periodic stimulation depend on the duration, frequency, and duty cycle of the stimulus.

### Modeling the response to temporally patterned stimulation

The response to the continuous stimulation suggested that input-output dynamics of the cellular responses could be modeled as a simple first-order function and that more complex stimulation programs could be understood within the same model framework. In the model, we assumed that the transport rates into and out of the nucleus are symmetric by the same process, although in reality inport and export of proteins are mediated by separate processes of different kinetics. Our model does not include photo-bleaching effects due to the use of confocal microscopy. We chose to model the GFP translocation response to continuous DEX stimulation as a first-order differential equation (See Materials and Methods; Figure S8). We used the same parameters that reproduced the response to continuous stimulation to model the translocation response to more complex frequency-dependent stimulation by 2 min-, 10 min-, and 40-min profiles with 50% duty cycle (Figure 4C; see Figure S5B). The simulated and observed responses reveal that the "input-output" response of a complex multicellular tissue to complex patterns of stimulation can be predicted by a systems-based model. Further analysis of the frequency response shows the logarithm of the frequency response of the magnitude of the output of intensity ratio divided by the input of DEX concentration with respect to the logarithm of the frequency of the stimulation (Figure S5C; see Materials and Methods). Transient responses between the “on” and “off” portions of the 2 min- and 10 min-periods lie below the temporal resolution of our experiments; however, the pattern of response is consistent with the signaling pathway that acts like a low-pass filter having a cut-off frequency of approximately 0.06 cycles per minute (Figure S5C). Thus, quantification and close examination of the systematic response to different stimulation programs (i.e. various “input” functions) highlights the biophysical processes that contributes to directing embryonic tissue responses to their complex chemical microenvironment.

## Discussion

Gradients of chemical factors drive emergent phenomena in embryos by stimulating cascades of cell signaling, gene regulatory networks, cell motility, and cell differentiation. Together, these cues provide positional information to establish distinct cell identities that self-assemble into functional tissues. As morphogenesis begins, gradients also provide instructive polarity cues telling cells their orientation within a field and providing guidance for directed cell rearrangement or movement. The extent and role of gradients *in vivo* continues to be debated [37]. By providing explicit spatial and temporal control over chemical gradients our study marks a key advance in studying the function of gradients as they interact with responsive embryonic tissues. In this paper we have integrated four key technologies to examine the role of gradients within developing embryonc tissues: 1) development of a sophisticated microfluidic system for the long-term precise production and control of spatial and temporal chemical gradients, 2) adaptation of *Xenopus* AC explants, a widely used embryonic tissue for signaling studies, that serves as a naive multicellular template for investigating cellular responses to chemical stimuli, 3) adaptation of a GFP-based biosensor and imaging techniques to visualize and quanitatively report cellular responses, and 4) development of mathematical models of chemical signaling within a living multicellular embryonic tissue.

Using these technologies we have investigated the stimulus-response function of a synthetic signaling network comprised of a microfluidically controlled hormone dexamethasone (DEX) and a protein-based biosensor that reports the level of dexamethasone by redistributing the biosensor (GR-nuc-GFP) from the cytoplasm to the nucleus. Studies with similar reporters [31], [36], [38] have reported that in the absence of DEX GFP fluorescence accumulates in the cytoplasm and that once DEX is added GFP is transported from the cytoplasm to the nucleus. Movement of GR-nuc-GFP into the nucleus in response to DEX is thought to depend on the removal of HSPs that bind the GR domain [39]. Maintenance of GR-nuc-GFP in the cytoplasm in the absence of DEX occurs due to "shielding" of the nuclear import signal by HSPs; after DEX is added HSPs are dislodged and GR-nuc-GFP is transported into the nucleus. Our first set of studies delivering constant DEX stimulation to AC explants expressing GR-nuc-GFP resulted in its rapid translocation into the nucleus with a half-time of ∼10 minutes similar to previous studies [31], [36], [38]. By controlling the timing and duty-cycle of DEX stimulation we found that tissues exposed to high levels of DEX at 50% duty cycle responded as if they had been exposed to 50% levels of DEX. We believe this was not due to dilution or mixing but rather due to the integrative nature of the DEX-GR signaling system.

Our approach combining spatiotemporally controlled stimulation technology and theoretical modeling allows us to probe the dynamic response of glucocorticoid receptor dynamics. Our results suggest that we might be able to explain the stimulus-response function of GR-nuc-GFP expressing multicellular tissues exposed to DEX with a set of first-order differential equations. Consider the biophysical and biochemical processes needed to move GFP into the nucleus: 1) microfluidic delivery of DEX to the tissues, 2) diffusion of DEX into the cytoplasm, 3) binding of DEX by the glucocorticoid receptor, 4) displacement of HSPs, 5) exposure of nuclear import signal, 6) recognition of the nuclear import signal by the import complex, and 7) translocation of GR-nuc-GFP into the nucleus. The delivery and diffusion of DEX into the cell are relatively very fast [40]. Translocation of GR-nuc-GFP into the nucleus is also expected to be very fast [41]. Thus, our study reports on the dynamics of DEX interactions with GR-nuc-GFP and the displacement of the HSPs. The transport rate will be essentially proportional to the cytoplasmic concentration of unshielded GR-nuc-GFP at the nuclear membrane. Since the transport rate is proportional to the concentration, the accumulation of GFP in the nucleus, i.e. the response, will be exponential. Using these equations we were able to capture the GFP response to a range of microfluidic stimulus frequencies. This approach provides a tool to investigate the design principles of signaling circuits and morphogenetic programs in developing embryonic tissues.

The cutoff frequency is a crucial characteristic of the frequency response as a signal filter; for instance, a cell response can be distinctly featured below and above this frequency such as the generation of repetitive ([Ca^2+^])-spikes varying in frequency, amplitude, and duration depending on the strength and type of the extracellular agonist [25]. In this study, the cell response can be discretely changed if the frequency of the periodic stimulation is lower than the cut-off frequency (16.6 min/cycle), while the cell response may be integrated if it is higher than the cut-off frequency, indicating that the response may reflect the concentration and duty cycle of the stimulation profile rather than it can be simply dictated by a single stimulus. In general, this filtering behavior means that cellular responses can differ when stimulated with the same concentration yet with different frequencies. The cut-off frequency responsible for this behavior can be attributed to the dynamic properties of signaling pathways; for example, cells may adjust a cutoff frequency (e.g. a tunable low-pass filter), filtering out high-frequency fluctuations or noise in signals and environmental cures [42]. In this way, cell responses with low-pass filters can make signaling cascades insensitive to noise and transient perturbations so that development can proceed without defects or consequences that can be caused by high-frequency extracellular perturbations.

The predictive spatiotemporal response of AC explants to the frequency modulated stimulation is a hallmark of a diverse array of biological and physical systems. Analysis of the response provides tremendous insight into fundamental emergent patterns that may evolve from complex systems. We have found that the dynamics of the "input-output" system with DEX and GR-nuc-GFP studied here resembles the dynamics of a resistor-capacitor network in that it has a well-defined input that is externally manipulatable and the response of the system to standard test inputs (e.g. step inputs or pulses) are useful for deducing parameters of the collective behavior. Our system provides the methodology for manipulating these biochemical inputs to examine and model the collective behavior of many biochemical reactions. This spatiotemporal approach along with a well documented modeling methodology has revealed an integrated signaling system in a developmental model tissue that we cannot only model, but one that also predicts frequency responses to time-varying stimuli. Thus, nanoscale molecular interactions in this multicellular developmental system result in highly regulated emergent behavior at size scales that are orders of magnitude larger, which we are able to determine experimentally by integrating systems biology and feedback microfluidic control approaches.

## Materials and Methods

### Ethics Statement

Animals used in this study were treated according to an animal use protocol (#0903349) reviewed and approved by the University of Pittsburgh Institutional Animal Care and Use Committee in order to meet all US government requirements.

### Dexamethasone Biosensor

We constructed a DEX biosensor (GR-nuc-GFP) by fusing the hormone binding domain of the human glucocorticoid receptor (GR) [29] in-frame to the 5′ end of a previously constructed nuclear localizing GFP (nuc-GFP) [30], [31] and confirmed the sequence of the resulting construct by sequencing. Early stage Xenopus embryos do not endogenously express hormone receptors and the concentrations of dexamethasone used here (25 µM) have no effect on normal development [29]. Capped mRNA encoding GR-nuc-GFP was synthesized and purified using standard methods from a linearized DNA template (AmpliCap Transcription kit; Epicentre Biotechnologies, Madison WI).

### Embryo handling, microsurgery and culture media

Eggs from female *Xenopus laevis* frogs were collected and fertilized *in vitro* following standard methods [43]. Fertilized embryos were dejellied in 2% Cysteine solution (pH 8) 30 min post fertilization. Embryos at the 2-cell stage were cultured in 3% Ficoll (Sigma, St. Louis MO) in 1× MBS (Modified Barth's solution) and microinjected with mRNA GR-nuc-GFP (0.2 ng). Embryos were cultured in 1/3× MBS to early gastrula stages [44]. Vitelline membranes were removed using forceps. Animal cap explants were microsurgically excised from stage 10 embryos using custom-made hair-loops and hair-knives in Danilchik's For Amy solution (DFA) with Bovine serum albumin (0.2% in media; Sigma-Aldrich) and antibiotic/antimycotic (0.8% in media; A5955, Sigma-Aldrich).

### Microscopy and image analysis

Explant attachment experiments were imaged with a digital charge-coupled device (CCD) camera (Scion Corp., Frederick, MD) mounted on a dissecting stereoscope. Microfluidic chambers with explants housed inside were placed on an x-y position controlled stage and time-lapse sequences for translocation experiments were recorded using a confocal scanning head (Leica TCS SP5: Leica Microsystems, Bannockburn IL) mounted on an inverted compound microscope. Still images and confocal sections were collected using a 0.7 N.A. 20X air or a 1.25 N.A. 40X oil-immersion objective. Confocal settings and adjustments were optimized for live-tissue imaging to reduce bleaching and maintain viability [45]. Time-lapse sequences were analyzed either manually or with custom-image processing macros (ImageJ, Wayne Rasband NIH). Projections of image stacks (50 sections at 0.2 µm intervals) were used to visualize nuclei and cytoplasm in the selected regions. Optical sectioning was needed since explants consist of cuboidal shaped cells from a couple of cell layers. In the confocal microscopic images, the data threshold was adjusted to better fit the dynamic range of the data (old range: 0–255, new range: 0-56) after quantification of intensity ratios.

### Microfluidic device design

Modular microfluidic devices were fabricated using standard soft-lithography techniques [46] with polydimethylsiloxane (PDMS) (SYLGARD 184, Dow Corning, Midland, MI). The microfluidic devices for two modular resistors (R20 and R60) were 20 and 60 mm long each and had cross sections 100 µm wide and 50 µm high. The microfluidic channel for the AC explants had three inlet channels that were rectangular cross sections with dimensions of 500 µm wide, 300 µm high, and 5 mm long. The central inlet was used as a temporary outlet for removing air bubbles in the fluidic network before the experiments began as these bubbles otherwise would shear tissue explants and disrupt the experiment. These inlet channels converged to form a single outlet channel (rectangular cross section 1500 µm wide, 300 µm high, and 10 mm long).

### Control system configuration

Microfluidic interface control system (Figure 1D) is composed of compressed nitrogen gas providing a constant pressure to the two reservoirs, one containing DFA, which flows through our pressure modulation system and the R20 fluidic resistance module before entering the microfluidic channel, and the other reservoir of 25 µM DEX diluted with DFA, passing through the R60 fluidic resistance module before entering the main microfluidic channel. The feedback loop modulates fluidic resistance and fluid volumes to regulate pressure at the channel inlet, which allows both long-term and high-speed control of the microfluidic interface. Microfluidic resistor modules were used to adjust an initial interface position at a defined location in the microfluidic channel.

### Computational fluid dynamics simulation

Numerical simulations of the flow field around the explants in the microfluidic channel were made using the commercial CFD solver, Fluent (ANSYS Inc., Lebanon, NH). The diffusion coefficients for the scalar species were specified to be 2.2E−10 m^2^/s corresponding to that of water at approximately room temperature [47]. The three dimensional computational domain was built using a structured hexahedral mesh with most of the cells having sides of 10 µm and four boundary layers (5∼10 µm) near the walls. Mesh independence was verified by examining higher density meshes. Flow rates were specified at the two inlets from the applied pressure in the experiments (Figure S4D). Atmospheric pressure was set at the outlet. The convergence limit was set so that velocities converged within 0.1% and mass fractions for scalar species reached their asymptotic values within 0.01%.

### Determination of model parameters

We chose to model this resposne as a chemical reaction of the GR-nuc-GFP system with DEX followed by the translocation into the nucleus. This model was represented by a first-order differential equation, which has been used in areas such as mathematical modeling of chemical reaction analysis [48]. In modeling, we employed a differential form 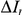 of the normalized intensity ratio for the initial value to be at the origin, although the normalized intensity ratio 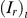 was used to compare experimental data to model values (Figure 4; Figure S5).
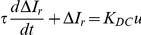
where 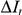 is the normalized intensity ratio differential (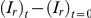) as a function of time, 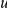 is the DEX concentration used, 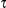 is the time constant of the equation, and 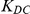 is the multiplicative constant that determines the steady-state value of the normalized intensity ratio differential. Model parameters 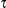 and 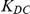 were determined using a least squares fitting to the continuous stimulation response. We obtained the response of the intensity ratio to a step input from the first-order differential equation model as a function of time. 
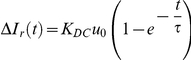
where 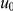 is the concentration of the applied dexamethasone as an input. From this equation, we obtained the following relation for 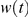.
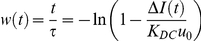



Then we applied a least square fitting to find the time constant 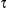 = 16.6 min. (R^2^ = 92.7) using Matlab (The MathWorks, Natick, MA) (Figure S8). The constant 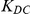 was determined by dividing the maximum value of the normalized intensity ratio differential by the concentration of DEX.
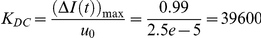



The transfer function *G(s)* with the input of DEX concentration and the output of the normalized intensity ratio differential and its magnitude were calculated as follows. 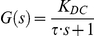


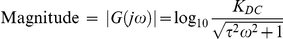



where 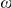 represented the frequency (cycle/min).

### Statistical analyses

Statistical analyses for verifying the significance of the intensity ratio values were carried out with the non-parametric Mann-Whitney U-test using commercial software, Minitab (Minitab Inc., State College, PA).

## Supporting Information

Figure S1 Effective biosensor exhibiting translocation into the nucleus after addition of DEX in multicellular embryonic tissues cultured in conventional chambers. (A-C) AC explants stimulated with DEX after (A) 0 min., (B) 60 min., and (C) 120 min. (D-F) Control AC explants separately cultured without DEX at (D) 0 min., (E) 60 min., and (F) 120 min. (G) Ratio of the intensity in the nucleus to the cytoplasm at 0 minutes, 60 minutes, and 120 minutes, which corresponds to the images (A)–(F). Error bars represent standard deviations for 20 cells.
(2.38 MB TIF)

Figure S2 Colocalization of propidium iodide stained DNA and GR-nuc-GFP in the nuclei of individual cells following DEX stimulation. The left panel shows colocalization of propidium iodide (red) and GR-nuc-GFP (green). The middle and right panels show propidium iodide and GR-nuc-GFP in grayscale, respectively.
(0.45 MB TIF)

Figure S3 Responses of individual cells to continuous DEX stimulation over time. Time-lapse confocal sequences of cells within AC explants expressing GR-nuc-GFP were collected over 60 minutes. Translocation of GFP into the nucleus was calculated from the ratio of GFP intensity within the nucleus and cytoplasm. The temporal profile was normalized to the ratio when DEX was first added.
(1.05 MB TIF)

Figure S4 CFD simulations depicting flow around the tissue explant in the microfluidic channel. (A) Diffusive dispersion through the channel at the AC explants at a flow rate of 30 µl/min. (B) Diffusion profile in the cross section to the downstream flow at flow rates of 10, 30, and 50 µl/min. These parameters include the need to prevent broad diffusive dispersion at low flow rates (A) and (B) as well as high shear forces that can detach explants at high flow rates. The lowest flow rate useable for our approach was determined using this CFD simulation while maintaining a diffusion thickness of less than 20 µm on the bottom plane at the end of the channel. (C) Diffusion thickness at different sections downstream at the middle layer relative to channel height. The determination of the diffusion thickness was based on a 10% threshold of mass fraction, which was normalized by the concentration across the interface. The determination of the diffusion thickness was based on a 10% threshold of mass fraction, which was normalized by the concentration across the interface. (D) Relative effects for flow rates, pressures, and Reynolds number. The red dashed box represents a useable range of the pressure in the experiment to prevent large diffusion and high shear stress based on the simulations. We then experimentally determined the highest flow rate possible for the experiment where the explants did not experience high shear force. An appropriate range of the flow rate was between 10 µl/min and 50 µl/min where the Reynolds number was less than 1. From these experimental and simulation results, we selected a flow rate of 30 µl/min for the experiment, which corresponded to an inlet pressure of 2 kPa. (E) Flow velocity and shear rates around the explant at a flow rate of 30 µl/min. This flow rate corresponded to a fluid velocity around the explant of less than 1.0 mm/s and a shear rate of less than 30 s–1.
(1.42 MB TIF)

Figure S5 Real and predicted frequency responses of individual embryonic cells from first-order differential equation model: simple modeling approach reveals emergent behaviors within complex embryonic system. (A) Responses of 30 individual tracked cells from 3 different tissue explants to four different stimulation cases with different duty cycles: CS (squares), 2-minute 50% duty cycle PS (triangles), 10-minute 50% duty cycle PS (circles), and 40-minute 50% duty cycle PS (diamonds). Error bars indicate standard deviations. (B) Using the data from CS results, a mathematical model was constructed using a first-order differential equation (see Materials and Methods). The parameters reproducing the response to CS were applied to the other PS cases to predict their response without any additional parameters (modeled CS, solid; modeled 2-minute PS, dashdot; modeled 10-minute PS, dotted; and modeled 40-minute PS, dashed). The modeled results closely approximate experimental results (CS, rectangles; 2-minute PS, triangles; 10-minute PS, circles; 40-minute PS, diamonds). (C) Frequency responses of three different PS profiles: 2-minute (0.5 cycle/min), 10-minute (0.1 cycle/min), and 40-minute (0.025 cycle/min). The lines come from the transfer function with a time constant of 16.6 minutes and the different constants for each stimulation case (see Materials and Methods; response in CS, square; response in 40-minute PS region, diamond).
(1.07 MB TIF)

Figure S6 Responses of AC explants to four different stimulation profiles. (A) Profiles of continuous stimulation (CS), and 50% duty cycle periodic stimulations (PS); 2 min-, 10 min-, and 40 min-period. (B) Responses of representative cells in AC explants with DEX (0 minutes, 30 minutes, and 60 minutes) and control regions without DEX (0 minutes, 30 minutes, and 60 minutes). (C) The ratio of the intensity in the nucleus to the cytoplasm at 0, 30, and 60 minutes. The scale bar is 20 µm. Error bars represent standard deviations for 20 cells sampled at each time step. (** indicates p<0.01).
(3.55 MB TIF)

Figure S7 GR-nuc-GFP can move out of the nucleus after DEX wash-out. The GR-complex can move out of the nucleus over a relatively longer period of time. We stimulated a tissue explant with a 20 minute pulse of DEX. We tracked and monitored 10 individual cells to observe GR-nuc-GFP translocation and obtain the intensity ratio after the DEX was washed out at 0 minutes. We calculated maximal projections of confocal stacks collected at (A) 10 min., (B) 20 min., and (C) 30 min. GFP intensity levels in the nucleus decrease over time. Error bars represent standard deviations.
(0.96 MB TIF)

Figure S8 Plot and formulas showing a least square fitting to find the time constant. The plot shows over time, which was obtained from the exponential function of the intensity ratio. We applied a least square fitting to find the linear slope from the plot and the time constant (see Materials and Methods; Determination of model parameters).
(0.04 MB TIF)

Video S1 AC tissue explant spreading in microfluidic channels. Tissue explants from different frogs attached to the substrate in the microfluidic channel spread out for 10 hours.
(0.77 MB MPG)
