# Supplementary figures and images for: Detection of Dynamic Spatiotemporal Response to Periodic Chemical Stimulation in a Xenopus Embryonic Tissue
Source: PLoS One. 2011 Jan 31;6(1):e14624. doi: 10.1371/journal.pone.0014624 (PMC3031512; doi:10.1371/journal.pone.0014624)

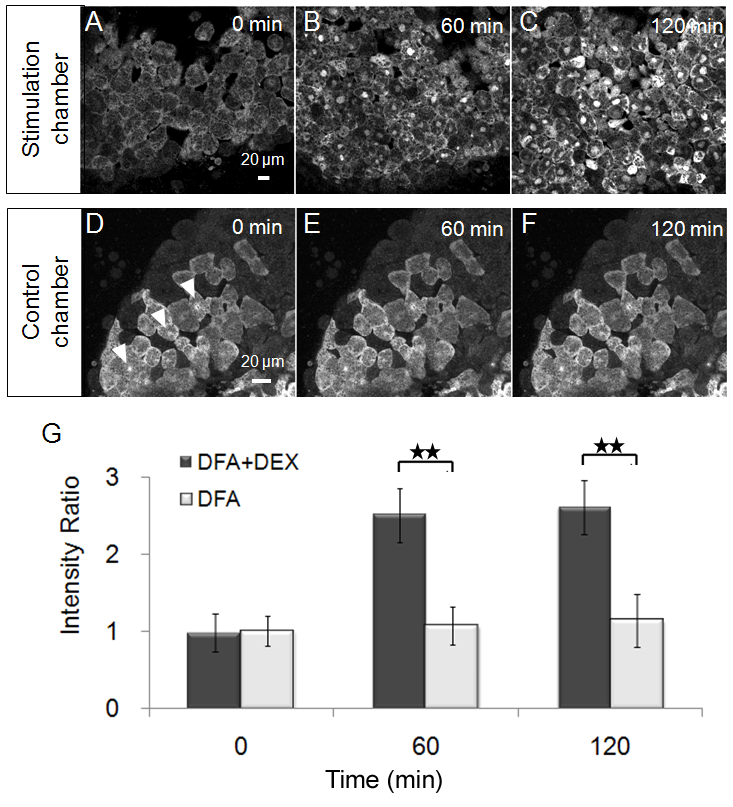

Supplement: Figure S1 — Effective biosensor exhibiting translocation into the nucleus after addition of DEX in multicellular embryonic tissues cultured in conventional chambers. (A-C) AC explants stimulated with DEX after (A) 0 min., (B) 60 min., and (C) 120 min. (D-F) Control AC explants separately cultured without DEX at (D) 0 min., (E) 60 min., and (F) 120 min. (G) Ratio of the intensity in the nucleus to the cytoplasm at 0 minutes, 60 minutes, and 120 minutes, which corresponds to the images (A)–(F). Error bars represent standard deviations for 20 cells. (2.38 MB TIF) [file pone.0014624.s001.tif]

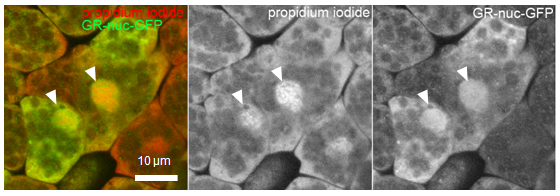

Supplement: Figure S2 — Colocalization of propidium iodide stained DNA and GR-nuc-GFP in the nuclei of individual cells following DEX stimulation. The left panel shows colocalization of propidium iodide (red) and GR-nuc-GFP (green). The middle and right panels show propidium iodide and GR-nuc-GFP in grayscale, respectively. (0.45 MB TIF) [file pone.0014624.s002.tif]

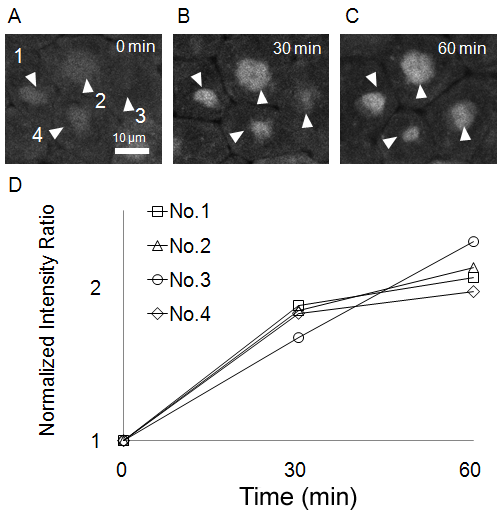

Supplement: Figure S3 — Responses of individual cells to continuous DEX stimulation over time. Time-lapse confocal sequences of cells within AC explants expressing GR-nuc-GFP were collected over 60 minutes. Translocation of GFP into the nucleus was calculated from the ratio of GFP intensity within the nucleus and cytoplasm. The temporal profile was normalized to the ratio when DEX was first added. (1.05 MB TIF) [file pone.0014624.s003.tif]

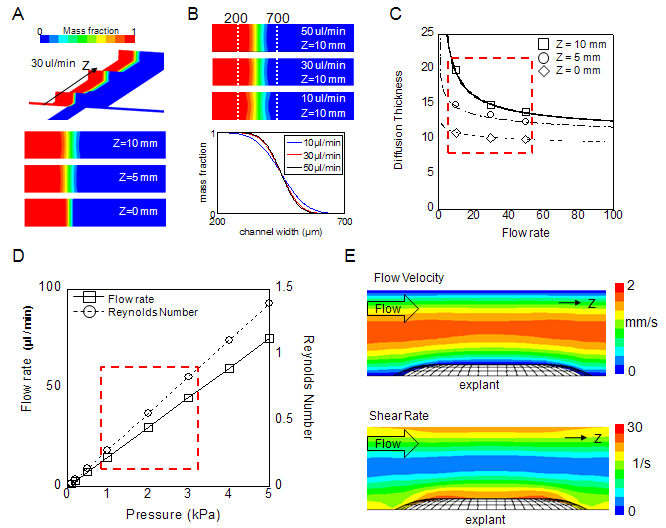

Supplement: Figure S4 — CFD simulations depicting flow around the tissue explant in the microfluidic channel. (A) Diffusive dispersion through the channel at the AC explants at a flow rate of 30 µl/min. (B) Diffusion profile in the cross section to the downstream flow at flow rates of 10, 30, and 50 µl/min. These parameters include the need to prevent broad diffusive dispersion at low flow rates (A) and (B) as well as high shear forces that can detach explants at high flow rates. The lowest flow rate useable for our approach was determined using this CFD simulation while maintaining a diffusion thickness of less than 20 µm on the bottom plane at the end of the channel. (C) Diffusion thickness at different sections downstream at the middle layer relative to channel height. The determination of the diffusion thickness was based on a 10% threshold of mass fraction, which was normalized by the concentration across the interface. The determination of the diffusion thickness was based on a 10% threshold of mass fraction, which was normalized by the concentration across the interface. (D) Relative effects for flow rates, pressures, and Reynolds number. The red dashed box represents a useable range of the pressure in the experiment to prevent large diffusion and high shear stress based on the simulations. We then experimentally determined the highest flow rate possible for the experiment where the explants did not experience high shear force. An appropriate range of the flow rate was between 10 µl/min and 50 µl/min where the Reynolds number was less than 1. From these experimental and simulation results, we selected a flow rate of 30 µl/min for the experiment, which corresponded to an inlet pressure of 2 kPa. (E) Flow velocity and shear rates around the explant at a flow rate of 30 µl/min. This flow rate corresponded to a fluid velocity around the explant of less than 1.0 mm/s and a shear rate of less than 30 s–1. (1.42 MB TIF) [file pone.0014624.s004.tif]

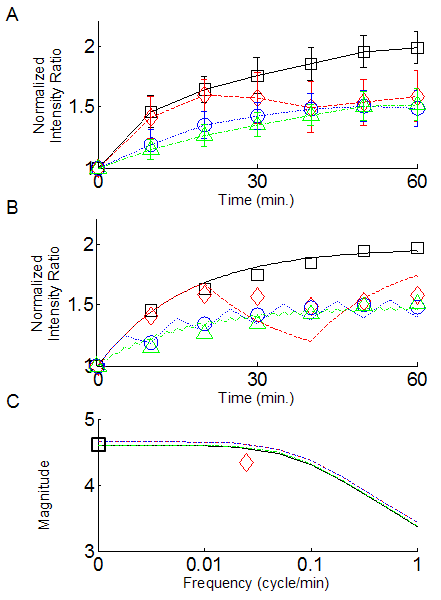

Supplement: Figure S5 — Real and predicted frequency responses of individual embryonic cells from first-order differential equation model: simple modeling approach reveals emergent behaviors within complex embryonic system. (A) Responses of 30 individual tracked cells from 3 different tissue explants to four different stimulation cases with different duty cycles: CS (squares), 2-minute 50% duty cycle PS (triangles), 10-minute 50% duty cycle PS (circles), and 40-minute 50% duty cycle PS (diamonds). Error bars indicate standard deviations. (B) Using the data from CS results, a mathematical model was constructed using a first-order differential equation (see Materials and Methods). The parameters reproducing the response to CS were applied to the other PS cases to predict their response without any additional parameters (modeled CS, solid; modeled 2-minute PS, dashdot; modeled 10-minute PS, dotted; and modeled 40-minute PS, dashed). The modeled results closely approximate experimental results (CS, rectangles; 2-minute PS, triangles; 10-minute PS, circles; 40-minute PS, diamonds). (C) Frequency responses of three different PS profiles: 2-minute (0.5 cycle/min), 10-minute (0.1 cycle/min), and 40-minute (0.025 cycle/min). The lines come from the transfer function with a time constant of 16.6 minutes and the different constants for each stimulation case (see Materials and Methods; response in CS, square; response in 40-minute PS region, diamond). (1.07 MB TIF) [file pone.0014624.s005.tif]

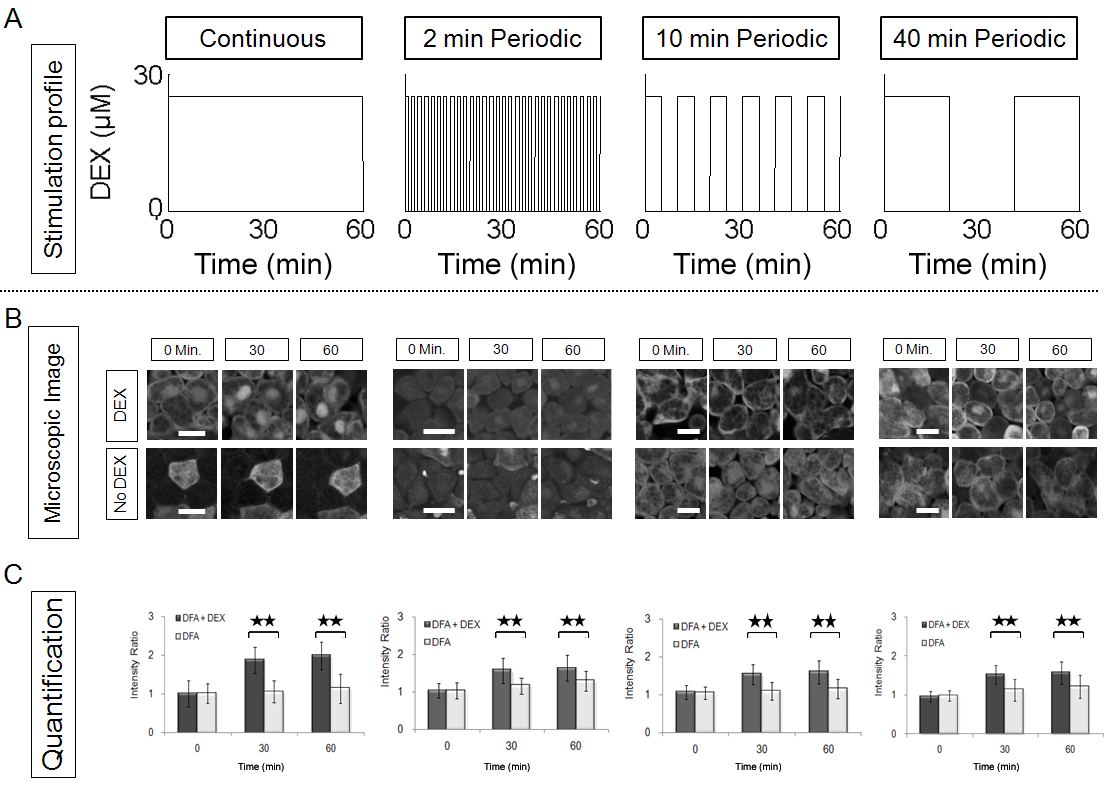

Supplement: Figure S6 — Responses of AC explants to four different stimulation profiles. (A) Profiles of continuous stimulation (CS), and 50% duty cycle periodic stimulations (PS); 2 min-, 10 min-, and 40 min-period. (B) Responses of representative cells in AC explants with DEX (0 minutes, 30 minutes, and 60 minutes) and control regions without DEX (0 minutes, 30 minutes, and 60 minutes). (C) The ratio of the intensity in the nucleus to the cytoplasm at 0, 30, and 60 minutes. The scale bar is 20 µm. Error bars represent standard deviations for 20 cells sampled at each time step. (** indicates p<0.01). (3.55 MB TIF) [file pone.0014624.s006.tif]

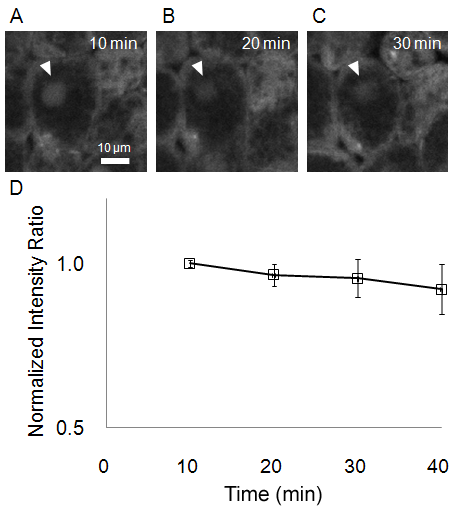

Supplement: Figure S7 — GR-nuc-GFP can move out of the nucleus after DEX wash-out. The GR-complex can move out of the nucleus over a relatively longer period of time. We stimulated a tissue explant with a 20 minute pulse of DEX. We tracked and monitored 10 individual cells to observe GR-nuc-GFP translocation and obtain the intensity ratio after the DEX was washed out at 0 minutes. We calculated maximal projections of confocal stacks collected at (A) 10 min., (B) 20 min., and (C) 30 min. GFP intensity levels in the nucleus decrease over time. Error bars represent standard deviations. (0.96 MB TIF) [file pone.0014624.s007.tif]

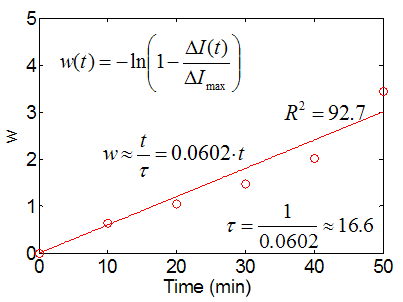

Supplement: Figure S8 — Plot and formulas showing a least square fitting to find the time constant. The plot shows over time, which was obtained from the exponential function of the intensity ratio. We applied a least square fitting to find the linear slope from the plot and the time constant (see Materials and Methods; Determination of model parameters). (0.04 MB TIF) [file pone.0014624.s008.tif]
